# Differential Impacts of Environmentally Relevant Microplastics on Gut Barrier Integrity in Mice Fed High-Fat Diet Versus Normal Chow Diet

**DOI:** 10.3390/metabo15080557

**Published:** 2025-08-20

**Authors:** Huixia Niu, Ying Yang, Yuting Zhou, Xue Ma, Zhehao Ding, Manjin Xu, Lizhi Wu, Xueqing Li, Mingluan Xing, Qin Zhang, Hao Chen, Xiongwei Tao, Zhe Mo, Zhijian Chen, Pengcheng Tu, Xiaoming Lou

**Affiliations:** 1 Center for Disease Control and Prevention of Jinyun County, 89 Cuizhu Road, Jinyun 321400, China; niuhuixia1232025@163.com (H.N.); yangying1232025@163.com (Y.Y.); zhangqin123202506@163.com (Q.Z.); chenhao123202506@163.com (H.C.); t173328120@163.com (X.T.); 2 Zhejiang Provincial Center for Disease Control and Prevention, 3399 Binsheng Road, Hangzhou 310051, China; zzzyt@stu.xmu.edu.cn (Y.Z.); xma@cdc.zj.cn (X.M.); 3190104398@zju.edu.cn (Z.D.); margexmj@outlook.com (M.X.); lzhwu@cdc.zj.cn (L.W.); xqli@cdc.zj.cn (X.L.); mlxing@cdc.zj.cn (M.X.); zhmo@cdc.zj.cn (Z.M.); zhjchen@cdc.zj.cn (Z.C.); 3 School of Medicine, Zhejiang University, 866 Yuhangtang Road, Hangzhou 310012, China

**Keywords:** emerging pollutants, microplastics, gut barrier, gut microbiota

## Abstract

Background: Despite escalating global pollution from microplastics (MPs) and the concurrent surge in high-fat food consumption, the health impacts of MP exposure on individuals under different dietary patterns remain poorly understood. Methods: This study investigated the differential effects of environmentally relevant concentrations of polystyrene microplastics (5 μm, 8 mg/kg) on gut barrier function in mice fed either a normal chow diet (CD) or a high-fat diet (HFD). Results: Key findings revealed that, in HFD-fed mice, MP exposure significantly reduced (*p* < 0.05) the transcriptional levels of genes encoding the tight junction proteins (ZO-1, Occludin, and Claudin-1), as well as the mucin protein Muc-2, accompanied by decreased protein expression levels of these markers in both colonic and ileal tissues. In contrast, no significant differences were observed in CD-fed mice exposed to MPs. Analysis of the gut microbiota and measurement of short-chain fatty acid (SCFA) metabolites showed that MPs induced significant alterations in the composition and diversity indices of the gut microbiota, along with a marked decrease (*p* < 0.05) in the levels of the characteristic metabolite butyrate in HFD-fed mice. Conversely, butyrate levels remained unchanged in CD-fed mice following MP exposure. Quantitative PCR (qPCR) and immunofluorescence staining of colonic tissues demonstrated that MP exposure significantly downregulated (*p* < 0.05) both the transcription and protein expression of peroxisome proliferator-activated receptor γ (PPARγ) in HFD-fed mice. Again, no significant changes were detected in CD-fed mice. Conclusions: These results collectively indicate that the impact of microplastics on the intestinal barrier differs significantly between mice fed normal and high-fat diets. The gut microbiota and its metabolites, particularly butyrate, may play a critical role, possibly through modulating PPARγ signaling. This study contributes valuable insights into understanding the toxicity profiles of microplastics and establishing crucial links between dietary patterns and the health effects of emerging pollutants.

## 1. Introduction

In 2004, Thompson et al. introduced the concept of microplastics, defining them as plastic particles with a diameter of less than 5 mm [1]. Microplastics (MPs) are ubiquitous in the environment and have been widely detected in food, water, and everyday consumer products [2,3,4,5,6]. These microplastics can enter the human body through ingestion, inhalation, and dermal contact. Studies indicate that ingestion is the primary route of exposure, with numerous investigations confirming the presence of microplastics in daily diets [7]. For instance, in 2015, Yang et al. detected microplastics in sea salt, lake salt, and rock/well salt [8]. Mattsson et al. found that microplastics in marine environments can transfer through the algae–daphnia–fish food chain [9]. Furthermore, microplastics can enter the human body through trophic transfer and nutritional uptake. Cauwenberghe et al. studied the potential risks of microplastics in seafood to humans, using mussels as a model to observe the direct impacts of microplastics via the food chain [10]. Their research revealed that consuming an average portion (250 g wet weight) of mussels could result in the ingestion of approximately 90 microplastic particles. Processed foods appear to contain higher levels of microplastics compared to unprocessed foods. Additionally, the presence of microplastics has been detected in human placenta and whole blood, suggesting that some inhaled microplastics are bioavailable: Microplastic particles are capable of being absorbed into the circulatory system, subsequently translocating to and accumulating within various organs [11,12]. Consequently, the potential health risks posed by microplastics have garnered increasing attention. Research has extensively examined the histopathological changes, oxidative stress, inflammatory responses, neurotoxicity, and reproductive toxicity induced by microplastics in biological systems. These include liver sinusoidal dilation, disordered hepatocyte arrangement, intestinal villi rupture, enterocyte lysis, elevated reactive oxygen species (ROS) levels, and upregulated expression of tumor necrosis factor alpha (TNF-α) and interleukin 6 (IL-6) [13,14,15,16,17].

The intestinal barrier function, comprising physical, chemical, immune, and microbial barriers, is critical in preventing the invasion of harmful substances from the gut and maintaining homeostasis between the internal and external environments [18]. The microbial barrier, in particular, consists of the microbiota that maintain cellular junctions and promote epithelial repair [19]. The human gut microbiota predominantly resides in the colon, with approximately 10^11^–10^12^ bacteria per gram of colonic tissue [20]. Changes in the composition and relative abundance of the gut microbiota can alter the metabolic products that enter the human body. Microbial-derived lipopolysaccharides (LPSs) and other endotoxins can leak into the bloodstream through a compromised gut barrier, leading to metabolic endotoxemia. LPS can reduce the expression of tight junction proteins between intestinal epithelial cells, increasing gut permeability. Conversely, short-chain fatty acids and indole metabolites produced by the gut microbiota can enhance the physical barrier of the gut by increasing the expression of tight junction proteins and cytoskeleton-associated proteins [21]. Studies have shown that sodium butyrate, within the concentration range of 1–10 mM, can increase Muc-2 protein levels, significantly improving epithelial function in human colonic epithelial cells [22]. Tang et al. also concluded that butyrate promotes gut barrier function [23]. Moreover, butyrate can bind to peroxisome proliferator-activated receptor γ (PPARγ), activating pathways that strengthen the gut barrier [24]. Simeoli et al. found that after administering Dextran Sulfate Sodium Salt (DSS) to mice for 7–12 days, PPARγ gene transcription levels significantly decreased, leading to impaired gut barrier function. Treatment with *Lactobacillus paracasei* improved gut barrier function and increased PPARγ gene transcription levels [25]. However, the effects of microplastic exposure on butyrate and PPARγ expression in colonic tissue and their roles in modulating gut barrier function remain to be further explored.

In recent years, the intake of high-fat foods worldwide has been steadily increasing. The potential health hazards of microplastics have attracted widespread attention both internationally and domestically. However, there is a lack of in-depth understanding of the differential health impacts of microplastic exposure under different dietary structures. Therefore, this study investigates the effects of environmentally relevant concentrations of microplastics on gut barrier function in mice fed either a normal or high-fat diet and examines whether these effects differ between the dietary groups. This research aims to enhance our understanding of the toxicity of environmental microplastics and their potential health impacts on humans.

## 2. Materials and Methods

### 2.1. Chemicals

Monodisperse polystyrene microspheres with a particle size of 5 μm were obtained from Tianjin BaseLine ChromTech Research Center (Tianjin, China). The stock solution (2.5% *w*/*v*) appeared as a milky-white suspension, with an initial concentration of 250 mg per 10 mL aqueous suspension. The characterized using a Coulter particle size analyzer [26]. The characterization of the polystyrene microplastics is presented in Supplementary Figure S1. Prior to use, the microplastic solution was diluted to a working concentration of 1.6 mg/mL using deionized water and stored at 4 °C for further experiments.

### 2.2. Animal and Experimental Scheme

Male C57BL/6 mice, aged 4–6 weeks, were procured from SLAC Laboratory Animal Co., Ltd. (Shanghai, China). All experimental procedures were conducted in accordance with protocols approved by the Animal Management and Ethics Committee of Zhejiang Chinese Medical University (No. 20230213-07). The mice were housed in an animal facility with controlled temperature (22 °C) and humidity (40–70%) levels, under a 12 h light/dark cycle. After a one-week acclimatization period, the mice were randomly divided into four groups (n = 10 per group; total N = 40) as follows [27,28]: (1) normal chow diet (CD) + water group (10 kcal%: fat-derived energy constitutes 10% of the total dietary energy in the feed), (2) CD + 8 mg/kg MP group (10 kcal%), (3) high-fat diet (HFD) + water group (60 kcal%: fat-derived energy constitutes 60% of the total dietary energy in the feed), and (4) HFD + 8 mg/kg MP group (60 kcal%).

Polystyrene microplastics with a particle size of 5 μm were selected for exposure at a dose of 8 mg/kg body weight per day. The particle size selection was based on the study by Jin et al. [27]. The exposure dose was chosen to simulate environmental microplastic concentrations. According to Deng et al., the estimated daily intake of microplastics for adults ranges from 13 to 39.3 mg/d [29]. Assuming an average adult body weight of 60 kg, the daily intake of microplastics per kg body weight is approximately 0.22–0.66 mg/kg. Using classical pharmacological and toxicological formulas, the estimated microplastic exposure dose for mice ranges from 2.7 to 8.1 mg/kg body weight [30]. Considering the daily food intake and average body weight of mice, the gavage dose in this study was set at 8 mg/kg body weight per day.

### 2.3. Tissue Collection and Histological Observation

Mice were fasted overnight and then euthanized by cervical dislocation [31]. Blood samples were collected prior to sacrifice. The serum was separated by centrifugation and stored at −80 °C for the subsequent analysis of inflammatory cytokine concentrations. Colonic tissues were collected, fixed in 4% paraformaldehyde, and embedded in paraffin for histopathological examination. Tissue sections (4 μm thick) were observed under an optical microscope. Another portion of the colonic tissue was immediately frozen in liquid nitrogen and stored at −80 °C until analysis.

Colonic tissue sections were subjected to hematoxylin and eosin (HE) staining, immunohistochemical staining, and immunofluorescence staining.

### 2.4. Biochemical Analysis

The levels of LPS, IL-1β, IL-10, TNF-α, and IL-6 in the serum were determined using enzyme-linked immunosorbent assay (ELISA) kits from Mei Mian Biotech Co., Ltd. (Yancheng, China).

### 2.5. qPCR Analysis

The remaining colonic tissue was used for qPCR analysis. Total RNA was extracted from the colonic tissue using a tissue homogenizer with TRIzol. The complementary DNA (cDNA) was synthesized using 4× genomic DNA (gDNA) wiper rMix and 5× HiScriptII qRT SuperMix II. Real-time PCR was performed with Forward Primer (10 μM), Reverse Primer (10 μM), 2× Taq Pro Universal SYBR qPCR Master Mix, and Template DNA/cDNA. Primer sequences are listed in Supplementary Table S1.

### 2.6. Gut Microbiota Analysis

Genomic DNA from fecal samples was extracted using the Mag-bind Soil DNA Kit. The V3-V4 region of the 16S ribosomal RNA (rRNA) of the gut microbiota was amplified using universal 16S rRNA primers. PCR products were recovered from 2% agarose gels, purified using the AxyPrep DNA Kit, and detected by 2% agarose electrophoresis. Library construction was performed using the TruSeq DNA PCR-Free Sample Preparation Kit. Sequencing was conducted on the Illumina NovaSeq 6000 platform (PE250). Operational taxonomic units (OTUs) with a similarity of over 97% were clustered, and the abundance of each OTU was statistically analyzed. The species composition and relative abundance of the gut microbiota at the phylum and genus levels were determined. Principal Coordinate Analysis (PCoA) was used to analyze community composition similarities and differences. Venn diagrams were used to count the shared and unique species among different experimental groups.

### 2.7. SCFAs Analysis

Metabolites were extracted using 20% phosphoric acid and 4-methylvaleric acid. Separation was carried out on an Agilent DB-FFAP capillary column (30 m × 250 μm × 0.25 μm) using a gas chromatography system. Fecal samples were detected in SCAN/SIM mode. The chromatographic peak areas were extracted using Mass Hunter software (MSD Chemstation Data Analysis 1701FA F.01.00) to construct standard curves, and the SCFA content in the samples was calculated based on these curves. The analysis of short-chain fatty acids (SCFAs) was performed by Shanghai Applied Protein Technology Co., Ltd. (Shanghai, China).

### 2.8. Statistical Analysis

Statistical analyses were performed using SPSS 22.0 (IBM, Inc., New York, NY, USA) and GraphPad Prism 8.4.2 software from GraphPad Software, version 8.4.2 for Windows, GraphPad Software, San Diego, CA, USA. Continuous data are presented as mean ± standard deviation ($\bar{x}$ ± SD). Between-group comparisons were analyzed by one-way ANOVA, with data transformation applied or non-parametric tests when normality assumptions were violated. All figures are two-tailed cutoffs, and significance was set at *p* < 0.05, unless otherwise stated.

## 3. Results

### 3.1. Levels of Inflammatory Factors and Barrier Protein Gene Transcription

**Figure 1:**
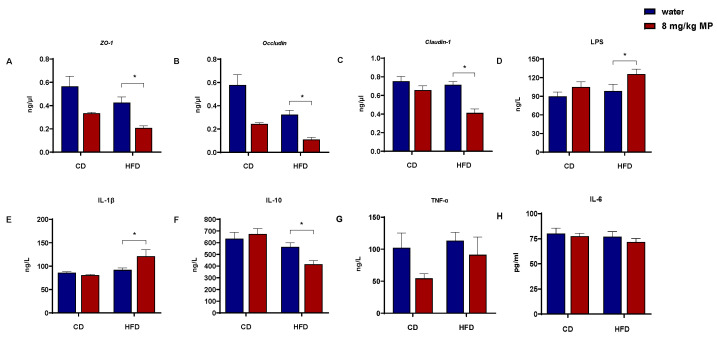
Polystyrene microplastic-induced changes in serum inflammatory factor and intestinal barrier protein gene expression of mice fed normal or high-fat diets: (**A**) transcription of ZO-1 proteins located in the colon, (**B**) transcription of Occludin proteins located in the colon, (**C**) transcription of Claudin-1 proteins located in the colon, (**D**) LPS content in mice serum, (**E**) IL-1β content in mice serum, (**F**) IL-10 content in mice serum, (**G**) TNF-α content in mice serum, and (**H**) IL-6 content in mice serum. * *p* < 0.05.

The intestinal barrier functions to segregate the gut microbiota and their metabolites from the host body. The physical barrier, composed of intestinal epithelial cells and the tight junction proteins that connect these cells, plays a critical role in preventing harmful substances from entering the body. To investigate the impact of polystyrene microplastic exposure on the transcription levels of tight junction proteins (ZO-1, Occludin, and Claudin-1) in the colonic tissue of mice, qPCR was performed (Figure 1A–C). Compared with the CD + water group, the HFD + water group showed significantly decreased transcription levels of ZO-1 and Occludin (*p* < 0.05), with no significant change in Claudin-1. The CD + 8 mg/kg MP group exhibited reduced transcription levels of ZO-1 and Occludin compared to the CD + water group (*p* < 0.05), with no significant change in Claudin-1. Additionally, the HFD + 8 mg/kg MP group demonstrated significantly reduced transcription levels of ZO-1, Occludin, and Claudin-1 compared to the HFD + water group (*p* < 0.05). These results indicate that microplastic exposure alone reduces the transcription levels of tight junction proteins in colonic tissue, and co-exposure with a high-fat diet exacerbates these effects.

Increased intestinal barrier permeability allows LPS and inflammatory factors from the gut to enter the circulatory system. ELISA kits were used to measure the levels of LPS and inflammatory factors in the serum of mice (Figure 1D–H). After 14 weeks of polystyrene microplastic exposure, serum IL-6 and TNF-α levels showed no significant changes. While IL-1β levels in the HFD + water group showed an increasing trend compared to the CD + water group, there was no significant statistical difference. There was no significant change in IL-1β levels between the CD + water and CD + 8 mg/kg MP groups. However, the HFD + 8 mg/kg MP group exhibited significantly elevated IL-1β levels compared to the HFD + water group (*p* < 0.05). For IL-10, the HFD + water group showed a decreasing trend compared to the CD + water group, with no significant difference between the CD + water and CD + 8 mg/kg MP groups. The HFD + 8 mg/kg MP group displayed significantly reduced IL-10 levels compared to the HFD + water group (*p* < 0.05). LPS levels showed an increasing trend in the HFD + water group compared to the CD + water group. Similar trends were observed in the CD + 8 mg/kg MP group compared to the CD + water group, with significantly elevated LPS levels in the HFD + 8 mg/kg MP group compared to the HFD + water group (*p* < 0.05).

### 3.2. Morphology in the Colon

**Figure 2:**
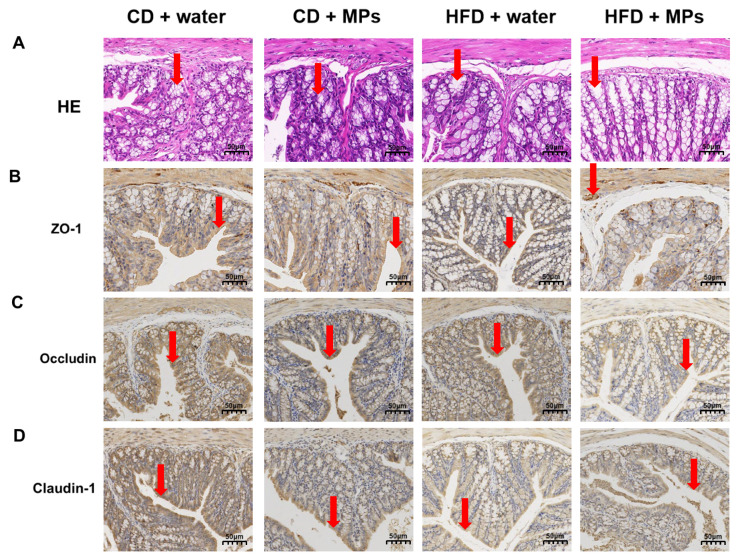
Polystyrene microplastic-induced changes in the colon morphology of mice fed normal or high-fat diets: (**A**) HE staining of colon tissue, (**B**) expression of ZO-1 proteins located in the colon, (**C**) expression of Occludin proteins located in the colon, and (**D**) expression of Claudin-1 proteins located in the colon. Red arrows: denote critical pathological features ((**A**) crypt structures; (**B**) ZO-1 protein expression; (**C**) Occludin protein expression; (**D**) Claudin-1 protein expression). Scale bar: 50 μm.

HE staining was performed to observe the morphological changes in the colonic tissue of mice following polystyrene microplastic exposure (Figure 2A). The results indicated that the colonic tissue of the CD + water group exhibited intact glands, clear crypt structures, regular arrangement, and continuous mucosal epithelium. There were no significant morphological changes in the colonic tissue of the CD + 8 mg/kg MP group compared to the control group, with normal tissue structure. However, the HFD + water group showed disrupted crypt structures, irregular crypt surfaces, and uneven mucosal surfaces. These effects were more pronounced in the HFD + 8 mg/kg MP group, indicating that microplastic exposure alone had no significant impact on colonic morphology, but co-exposure with a high-fat diet exacerbated the morphological changes induced by the high-fat diet. Immunohistochemical staining was performed to observe the expression of tight junction proteins in the colonic tissue following polystyrene microplastic exposure (Figure 2B–D). The CD + water group showed uniform and continuous positive brown areas on the surface of colonic epithelial cells, with no significant differences in the CD + 8 mg/kg MP group. The HFD + water group exhibited discontinuous and diffuse distribution, with significantly reduced expression areas of ZO-1, Occludin, and Claudin-1 proteins in the HFD + 8 mg/kg MP group. These results suggest that, while microplastic exposure alone does not significantly affect the expression of tight junction proteins, co-exposure with a high-fat diet exacerbates the impact of the high-fat diet on tight junction protein expression.

### 3.3. Composition and Diversity of the Gut Microbiota

**Figure 3:**
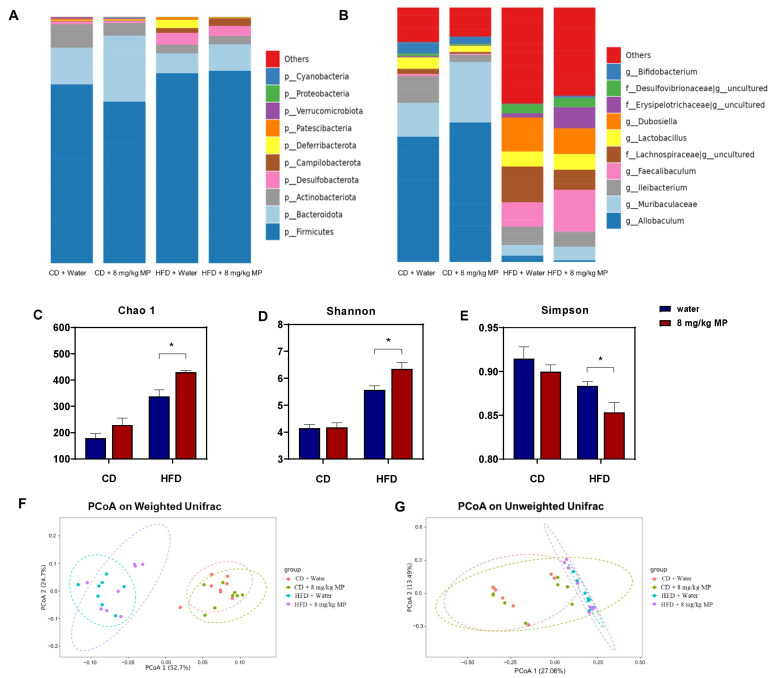
Polystyrene microplastic-induced changes in the composition and diversity of gut microbiota of mice fed normal or high-fat diets (**A**) Changes in gut microbiota composition at the phylum level. (**B**) Changes in gut microbiota composition at the genus level. (**C**) The Chao 1 index analyzes changes in gut microbiota diversity in mice. (**D**) The Shannon index analyzes changes in gut microbiota diversity in mice. (**E**) The Simpson index analyzes changes in gut microbiota diversity in mice. (**F**) Changes in gut microflora diversity in mice analyzed by Principal Coordinate Analysis (PCoA) based on weighted UniFrac distance. (**G**) Changes in gut microflora diversity in mice analyzed by PCoA based on unweighted UniFrac distance. * *p* < 0.05.

The gut microbiota plays a vital role in maintaining host metabolism and health and regulating intestinal barrier function. To investigate changes in the gut microbiota composition following polystyrene microplastic exposure, the composition at the phylum level was analyzed based on the 16S rRNA sequencing results (Figure 3A). The results showed that *Firmicutes*, *Bacteroidota*, *Actinobacteriota*, *Desulfobacterota*, *Campilobacterota*, and *Deferribacterota* were the most abundant phyla in the mouse gut microbiota. The HFD + water group exhibited significantly increased levels of *Firmicutes*, *Desulfobacterota*, and *Campilobacterota* and decreased levels of *Bacteroidota* and *Actinobacteriota* compared to the CD + water group. The CD + 8 mg/kg MP group also showed increased relative abundance of *Firmicutes*, *Desulfobacterota*, and *Campilobacterota*, with a decreasing trend in the abundance of *Bacteroidota* and *Actinobacteriota* compared to the CD + water group. Similar trends were observed in the HFD + 8 mg/kg MP group compared to the HFD + water group.

To reveal more detailed changes in the gut microbiota composition, genus-level analysis was performed (Figure 3B). The most abundant genera included *Allobaculum*, *Muribaculaceae*, *Ileibacterium*, *Faecalibaculum*, *Lachnospiraceae*, *Lactobacillus*, and *Dubosiella*. Compared to the CD + water group, the HFD + water group showed significantly increased levels of *Faecalibaculum*, *Lachnospiraceae*, *Lactobacillus*, and *Dubosiella*, with decreased relative abundance of *Allobaculum* and *Muribaculaceae*. The HFD + 8 mg/kg MP group exhibited similar trends compared to the HFD + water group.

Previous studies have shown that the diversity of the gut microbiota can be influenced by environmental chemicals. Analysis of the 16S rRNA sequencing results revealed the impact of microplastic exposure on the diversity of the gut microbiota (Figure 3C–G). The Chao 1 index, which estimates species richness, was significantly higher in the HFD + water group compared to the CD + water group (*p* < 0.05). While there was no significant difference between the CD + 8 mg/kg MP group and the CD + water group, an increasing trend in the index values was observed. The HFD + 8 mg/kg MP group showed significantly higher Chao 1 indices compared to the HFD + water group (*p* < 0.05). The Shannon and Simpson indices, which estimate microbial diversity, indicated increased diversity when the Shannon index was high and the Simpson index was low. The HFD + water group showed significantly increased Shannon indices and decreased Simpson indices compared to the CD + water group (*p* < 0.05). There were no significant differences between the CD + 8 mg/kg MP group and the CD + water group, but the HFD + 8 mg/kg MP group exhibited increased Shannon indices and decreased Simpson indices compared to the HFD + water group (*p* < 0.05). These results suggest that polystyrene microplastic exposure significantly increases the α-diversity of the gut microbiota.

Principal Coordinate Analysis (PCoA) of the 16S rRNA sequencing results was performed. Based on the weighted UniFrac distance algorithm, there were significant differences in the OTU levels between normal-diet-fed mice and high-fat-diet-fed mice, with no overlap. Microplastic-exposed mice and control mice showed significant differences in OTU levels regardless of diet, with more pronounced differences in the high-fat-diet-fed mice. The unweighted UniFrac distance algorithm also revealed significant differences in OTU levels between normal-diet-fed and high-fat-diet-fed mice, with minimal overlap. Significant differences in OTU levels were observed between microplastic-exposed and control mice regardless of diet, with more pronounced differences in high-fat-diet-fed mice. These results indicate that polystyrene microplastic exposure significantly increases the β-diversity of the gut microbiota.

### 3.4. Changes in the Gut Microbiota

**Figure 4:**
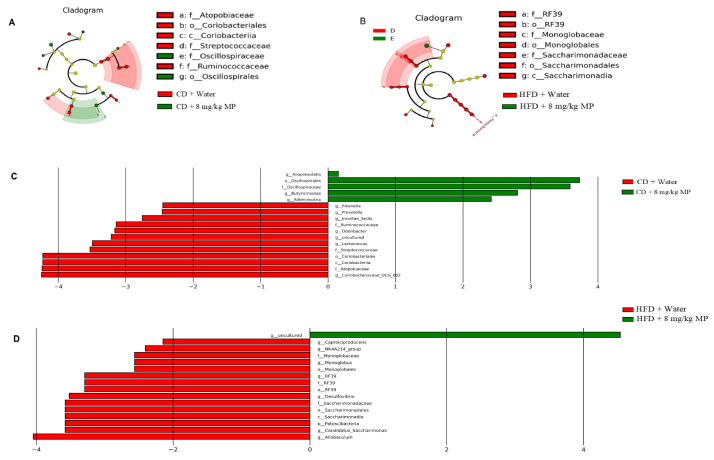
Polystyrene microplastic-induced changes in gut microbiota composition of mice fed normal or high-fat diets: the differences in microbiota between groups after exposure to polystyrene microplastics are shown by LEfSe analysis. (**A**) Cladogram analysis of the gut microbiota of mice in CD + water and CD + 8 mg/kg MP groups. (**B**) Histogram of the distribution of linear discriminant analysis (LDA) values of the gut microbiota of mice in CD + water and CD + 8 mg/kg MP groups. (**C**) Cladogram analysis of the gut microbiota of mice in HFD + water and HFD + 8 mg/kg MP groups. (**D**) Histogram of the distribution of LDA values of the gut microbiota of mice in HFD + water and HFD + 8 mg/kg MP groups.

To further assess the impact of polystyrene microplastic exposure on the composition of the gut microbiota in mice, LEfSe analysis was used to identify specific changes. Compared to the CD + water group, the relative abundances of *g__Allobaculum*, *o__Oscillospirales*, *f__Oscillospiraceae*, *g__Butyricimonas*, and *g__Adlercreutzia* significantly increased in the CD + 8 mg/kg MP group after polystyrene microplastic exposure. In comparison to the HFD + water group, the relative abundance of *g__uncultured* significantly increased in the HFD + 8 mg/kg MP group. These results indicate that exposure to microplastics significantly alters the composition of the gut microbiota in mice (Figure 4).

### 3.5. Short-Chain Fatty Acids

**Figure 5:**
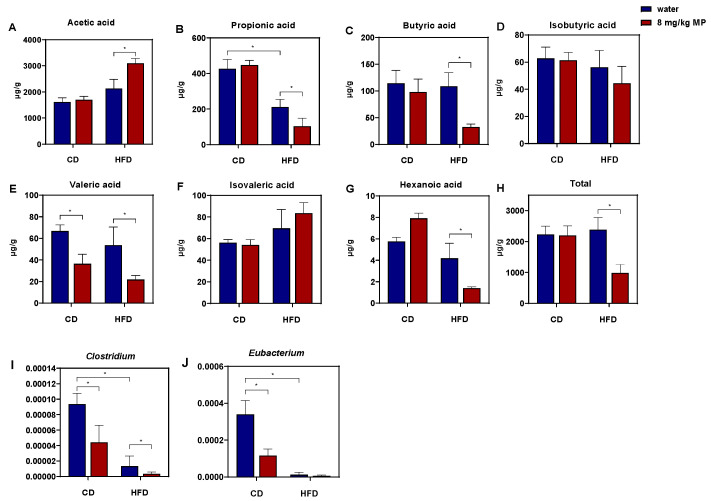
Polystyrene microplastic-induced changes in fecal short-chain fatty acid and butyrate-producing gut microbiota of mice fed normal chow diets (CDs) or high-fat diets (HFD). (**A**) Acetic acid in mouse feces. (**B**) Propionic acid in mouse feces. (**C**) Butyric acid in mouse feces. (**D**) Isobutyric acid in mouse feces. (**E**) Valeric acid in mouse feces. (**F**) Isovaleric acid in mouse feces. (**G**) Hexanoic acid in mouse feces. (**H**) Total short-chain fatty acid in mouse feces. (**I**) Changes in relative abundance of *Clostridium*. (**J**) Changes in relative abundance of *Eubacterium*. * *p* < 0.05.

Due to the close relationship between short-chain fatty acids (SCFAs) and the intestinal barrier, changes in the content of SCFAs in fecal samples were analyzed after microplastic exposure, as shown in Figure 5A–H. Analysis revealed no significant difference in total SCFA content between the CD + water group and the HFD + water group, nor between the CD + water group and the CD + 8 mg/kg MP group. However, compared to the HFD + water group, the total SCFA content significantly decreased in the HFD + 8 mg/kg MP group (*p* < 0.05). Although differences in specific SCFA contents between the CD + water group and the HFD + water group were not statistically significant, propionate, valerate, and caproate showed a decreasing trend, while acetate and isovalerate exhibited an increasing trend. Compared to the CD + water group, the CD + 8 mg/kg MP group showed a significant decrease in valerate, but other SCFAs showed only changing trends without statistical significance. Compared to the HFD + water group, the HFD + 8 mg/kg MP group showed no significant differences in isobutyrate and isovalerate but had significantly increased acetate and significantly decreased propionate, butyrate, valerate, and caproate (*p* < 0.05). These results suggest that polystyrene microplastic exposure alone does not significantly affect SCFA metabolism, but combined exposure with a high-fat diet exacerbates the impact on SCFA metabolism.

Butyrate may affect intestinal barrier function by binding to PPARγ, so this study aims to explore whether the impact of polystyrene microplastic exposure on glucose metabolism in mice is related to intestinal barrier dysfunction caused by reduced butyrate levels. According to the above results, the butyrate content in fecal samples significantly decreased in the HFD + 8 mg/kg MP group compared to the HFD + water group. Therefore, quantitative analysis of butyrate-producing bacteria in fecal samples was conducted, as shown in Figure 5I,J. The results showed that, compared to the CD + water group, the relative abundances of *Clostridium* and *Eubacterium* significantly decreased in the fecal samples of the CD + 8 mg/kg MP group (*p* < 0.05). Moreover, compared to the HFD + water group, the relative abundances of *Clostridium* and *Eubacterium* further decreased in the HFD + 8 mg/kg MP group. These results indicate that polystyrene microplastics affect the metabolic pathway of butyrate by reducing the relative abundance of butyrate-producing bacteria in the gut microbiota of mice, ultimately leading to a decrease in butyrate content.

### 3.6. Expression of PPARγ

**Figure 6:**
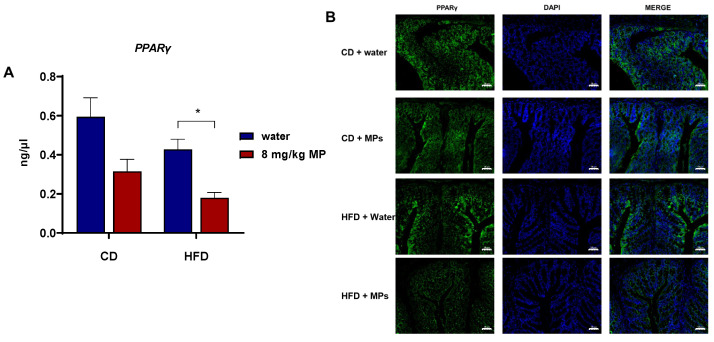
Polystyrene microplastic-induced changes in PPARγ expression levels in the colon tissue of mice fed normal or high-fat diets (**A**) qPCR analysis of PPARγ gene transcription. (**B**) Immunofluorescence staining of PPARγ protein expression (CD + water: normal chow diet + water group; CD + MPs: normal chow diet + 8 mg/kg MP group; HFD + water: high-fat diet + water group; HFD + MPs: high-fat diet + 8 mg/kg MP group). Scale bar: 50 μm, * *p* < 0.05.

The nuclear transcription factor PPARγ binds to butyrate and participates in the regulation of intestinal barrier function. Therefore, qPCR was used to observe the transcription of the PPARγ gene in the colon tissues of mice after polystyrene microplastic exposure, as shown in Figure 6A. Compared to the CD + water group, the transcription level of the PPARγ gene significantly decreased in the colon tissues of the CD + 8 mg/kg MP group (*p* < 0.05). Similarly, compared to the HFD + water group, the transcription level of the PPARγ gene significantly decreased in the HFD + 8 mg/kg MP group (*p* < 0.05). These results indicate that microplastic exposure significantly reduces the transcription level of the PPARγ gene in colon tissues, and when combined with a high-fat diet, microplastic exposure exacerbates the impact on the transcription level of the PPARγ gene.

Immunofluorescence staining of PPARγ protein in colon tissues was used to observe the effect of polystyrene microplastic exposure on the expression of the PPARγ gene in colon tissues. The results showed that compared to the CD + water group, the expression of PPARγ significantly decreased in the HFD + water group. There was no significant difference in the expression of PPARγ in colon tissues between the CD + water group and the CD + 8 mg/kg MP group. However, compared to the HFD + water group, the expression of PPARγ decreased even more significantly in the HFD + 8 mg/kg MP group. These results indicate that microplastic exposure alone does not significantly affect the expression of PPARγ in colon tissues, but when combined with a high-fat diet, microplastic exposure exacerbates the impact on the expression of PPARγ in the colon tissues of mice.

## 4. Discussion

Human activities have led to the pervasive presence of microplastics in the environment, which can enter the human body through ingestion, inhalation, and dermal contact. Consequently, increasing research attention has been directed towards the potential hazards posed to human health by microplastic exposure. Given the ubiquitous presence of microplastics and high-fat diets in human life, this study utilizes mice fed normal and high-fat diets to investigate the effects of microplastic exposure on gut barrier function, as well as the differential impacts of these dietary patterns. Our findings indicate that environmental concentrations of microplastic exposure exacerbate high-fat-diet-induced gut barrier dysfunction and increased intestinal permeability. The underlying mechanism may involve microplastic-induced gut dysbiosis, significant reductions in the relative abundance of the butyrate-producing bacteria *Clostridium* and *Eubacterium*, decreased butyrate levels, and reduced PPARγ receptor expression, ultimately leading to impaired gut barrier function.

Organisms are continually exposed to microplastics, with humans facing complex and varied exposure pathways that make intake assessments highly variable [32]. In this study, we selected a microplastic exposure dose of 8 mg/kg body weight/day, derived from environmental microplastic concentrations converted using classical pharmacological and toxicological formulas. Our findings indicate that although the high-fat diet mice did not reach statistical significance, they still exhibited a discernible trend of change. Similarly, numerous studies have demonstrated that dietary fat can directly modulate the structural integrity of the intestinal barrier, thereby influencing intestinal permeability. For instance, long-term high-fat diet feeding has been shown to downregulate the expression of genes encoding tight junction proteins [33]. Kirpich et al. reported that continuous feeding of a diet rich in unsaturated fatty acids significantly reduced the expression of tight junction proteins and increased the flux of fluorescein isothiocyanate (FITC)-conjugated 4 kDa dextran [34]. Combined exposure to a high-fat diet and microplastics results in the significantly reduced expression of tight junction proteins (ZO-1, Occludin, and Claudin-1) in the colonic tissues of mice, leading to disrupted and diffuse distribution and impaired physical gut barriers. The results indicate that microplastics may exacerbate intestinal barrier dysfunction induced by a high-fat diet. Yan et al. similarly observed that polystyrene microplastic exposure in rats resulted in shallower crypt structures and damaged mucosal barriers. Additionally, gut permeability analysis revealed significantly elevated plasma D-lactate and diamine oxidase (DAO) levels in microplastic-exposed mice, biomarkers indicative of leaky gut syndrome [35]. These findings were corroborated by Okamura’s experiments, where fluorescein isothiocyanate (FITC)-dextran assay results demonstrated higher green fluorescent protein (GFP) signals in the high-fat diet and microplastic-exposed rats compared to the high-fat diet control group [36]. In our study, microplastics may affect people on a high-fat diet more than those on a normal diet, which may be attributed to the synergistic effects between microplastics and a high-fat diet upon co-exposure: specifically, the combined impact of HFD and microplastics exceeds the sum of their individual effects, thereby exacerbating the influence of HFD on intestinal barrier function and gut microbiota in mice. The lack of significant effects of microplastic exposure on mice under a normal diet in this study may be attributed to the selected particle size, dosage, or duration of exposure. However, upon retrospective analysis of the results, we observed that although the differences were not statistically significant, normal-diet mice exposed to microplastics exhibited trends consistent with those observed in high-fat-diet mice.

The disruption of tight junctions increases intestinal permeability, allowing harmful substances such as bacteria and endotoxins to enter systemic circulation, adversely affecting organismal health [37,38]. Serum analysis revealed elevated levels of LPS and the pro-inflammatory cytokine IL-1β, along with decreased levels of the anti-inflammatory cytokine IL-10 in the HFD + 8 mg/kg MP group compared to the HFD + water group. LPS, a major component of the outer membrane of Gram-negative bacteria, showed increased relative abundance in response to microplastic exposure, significantly elevating endotoxin levels in the serum and indicating compromised gut barrier function. In the bloodstream, LPS forms trimers with lipopolysaccharide-binding protein, signaling through Toll-like receptors and pattern recognition receptors to activate multiple inflammatory pathways and promote chronic systemic inflammation [39,40].

The gut microbiota plays a crucial role in maintaining gut barrier function, making it essential to examine the impact of microplastic exposure on the gut microbiota. While no significant differences were observed in alpha diversity between the normal diet microplastic group and the normal diet control group, combined exposure to a high-fat diet and microplastics resulted in increased alpha diversity and significant differences in OTU levels between exposed and control mice. These findings are consistent with numerous studies indicating that microplastic exposure disrupts gut microbiota diversity and composition. For instance, Marine medaka (*Oryzias melastigma*) exposed to 50 nm and 45 μm polystyrene microplastics exhibited altered gut microbiota diversity and composition, with increased alpha diversity and changes in principal components [41]. Jin et al. also reported significant alterations in gut microbiota composition at the genus level, with 15 bacterial taxa showing notable changes following microplastic exposure [27]. Li’s mouse model study demonstrated that environmental microplastic exposure (6, 15, 60, and 600 μg/d) increases total gut microbiota, bacterial abundance, and diversity at high concentrations [14]. In contrast to control mice, exposed mice showed a significant increase in *Staphylococcus* and a decrease in *Parabacteroides* relative abundance. Another study found that exposure to 0.5 μm and 50 μm polystyrene microplastics significantly reduced gut microbiota diversity, with 310 OTUs affected at the phylum level, and notable reductions in fecal Firmicutes and α-Proteobacteria relative abundance in the 0.5 μm exposure group [42]. However, the authors did not provide a detailed explanation of the specific mechanisms underlying the reduction in gut microbiota diversity following microplastic exposure in the referenced literature. We speculate that this phenomenon may be associated with microplastic-induced damage to the intestinal physical barrier and mucus layer, oxidative stress and inflammatory responses, direct toxicity to gut microbiota, as well as alterations in intestinal physiology and function. Regarding the observed increase in gut microbiota diversity in the high-fat diet + microplastic group in our study, a plausible explanation is that the co-exposure to microplastics and a high-fat diet may lead to an elevated diversity of certain harmful bacterial species in the gut microbiota of mice, ultimately contributing to an overall increase in microbial diversity. Li et al. also reported microplastic-induced gut dysbiosis associated with intestinal inflammation in mice: The treatment of microplastics can significantly increase the relative abundance of *Staphylococcus* genera in the gut microbiota of mice, and the upregulation of *Staphylococcus* abundance may potentially induce an elevation in IL-1α levels [14]. Furthermore, another study has demonstrated that *Staphylococcus aureus* infection can induce the expression of the pro-inflammatory cytokine IL-1α [43].

Butyrate, a critical short-chain fatty acid, binds to PPARγ receptors in the gut to activate pathways that regulate gut barrier function [25,44]. In this study, combined exposure to a high-fat diet and microplastics resulted in significantly decreased butyrate levels and a reduced relative abundance of butyrate-producing bacteria. The expression of PPARγ in colonic tissues mirrored the trend in butyrate levels, with significantly decreased transcription and expression in the HFD + 8 mg/kg MP group compared to the HFD + water group. Okamura similarly concluded that short-chain fatty acid concentrations in fecal samples were significantly lower in the high-fat diet and microplastic-exposed mice compared to high-fat diet controls [36]. Kundu’s study demonstrated that gavage with *Salmonella typhimurium* suppressed PPARγ expression in gut epithelial cells, inducing acute infectious colitis [45]. Exposure to DSS also significantly reduced PPARγ transcription levels and impaired gut barrier function [25]. These results suggest that butyrate production enhances gut barrier function by binding to PPARγ receptors, mitigating microplastic-induced intestinal inflammation.

## 5. Conclusions

This study demonstrates that environmental concentrations of microplastic exposure can induce gut dysbiosis, alter butyrate levels, and reduce PPARγ receptor expression, leading to gut barrier dysfunction in mice. Furthermore, the impact of microplastic exposure on gut barrier function varies with different dietary structures. In high-fat diet-fed mice, the co-exposure to microplastics was particularly notable, exacerbating the adverse effects on the gut microbiota, butyrate levels, and intestinal barrier function. Considering the unavoidable human exposure to microplastics and the critical relationship between gut barrier function and human health, these findings contribute to a better understanding of the health effects of microplastics and their toxicity in relation to dietary structures.

## Data Availability

3 Data available on request due to restrictions eg privacy or ethical. The data presented in this study are available on request from the corresponding author. The data are not publicly available due to institutional animal ethics committee restrictions requiring controlled access to primary experimental datasets involving vertebrate animals (Approval No. 20230213-07).

## Supplementary Materials

The following supporting information can be downloaded at https://www.mdpi.com/article/10.3390/metabo15080557/s1, Figure S1: Coulter particle size analysis of polystyrene microspheres (offered by Tianjin BaseLine ChromTech Research Center); Table S1: Primer sequence list.

## Abbreviations

The following abbreviations are used in this manuscript:

MPs	Microplastics
ROS	Reactive oxygen species
CD	Chow diet
HFD	High-fat diet
PPARγ	Peroxisome proliferator-activated receptor γ
LPS	Lipopolysaccharides
ELISA	Enzyme-linked immunosorbent assay
PCoA	Principal Coordinate Analysis
SCFAs	Short-chain fatty acids
