# Supplementary material for: Differential Impacts of Environmentally Relevant Microplastics on Gut Barrier Integrity in Mice Fed High-Fat Diet Versus Normal Chow Diet
Source: Metabolites. 2025 Aug 20;15(8):557. doi: 10.3390/metabo15080557 (PMC12388625; doi:10.3390/metabo15080557)
Supplement: Supplementary file 1 [file metabolites-15-00557-s001.zip › metabolites-3758167-supplementary.pdf]

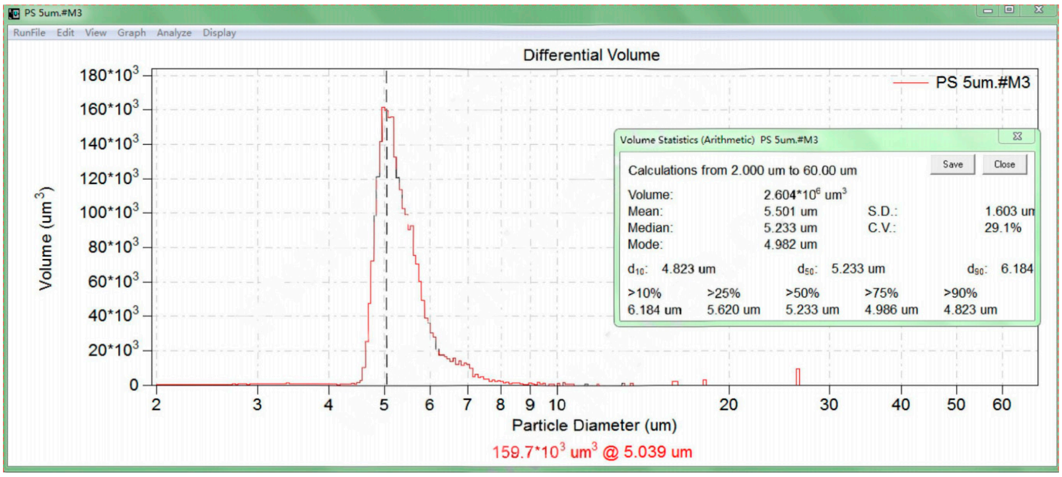

Figure S1. Coulter particle size analysis of polystyrene microspheres (Offered by Tianjin BaseLine ChromTech Research Center)

Table S1. Primer Sequence List

Gene	Primer	Python (5'-3')	Length
Mus GAPDH	Forward	ATGGGTGTGAACCACGAGA	19 bp
	Reverse	CAGGGATGATGTTCTGGGCA	
Mus PPARγ	Forward	GTGGGGATGTCTCACAATGC	20 bp
	Reverse	TTTCCTGTCAAGATCGCCCT	
Mus claudin-1	Forward	CCCCATCAATGCCAGGTATG	20 bp
	Reverse	GGTGTTGGCTTGGGATAAGG	
Mus Zo1	Forward	AGGACACCAAAGCATGTGAG	20 bp
	Reverse	GGCATTCCTGCTGGTTACA	
Mus Occludin	Forward	GTGAGCTGTGATGTGTGTTGAGCT	24 bp
	Reverse	GTGGGGAACGTGGCCGATATAATG	
